# Author Correction: Biodegradable black phosphorus-based nanospheres for in vivo photothermal cancer therapy

**DOI:** 10.1038/s41467-021-23210-z

**Published:** 2021-06-18

**Authors:** Jundong Shao, Hanhan Xie, Hao Huang, Zhibin Li, Zhengbo Sun, Yanhua Xu, Quanlan Xiao, Xue-Feng Yu, Yuetao Zhao, Han Zhang, Huaiyu Wang, Paul K. Chu

**Affiliations:** 1 Institute of Biomedicine and Biotechnology, Shenzhen Institutes of Advanced Technology, Chinese Academy of Sciences, Shenzhen, 518055 China; 2 SZU-NUS Collaborative Innovation Center for Optoelectronic Science and Technology, and Key Laboratory of Optoelectronic Devices and Systems of Ministry of Education and Guangdong Province, College of Optoelectronic Engineering, Shenzhen University, Shenzhen, 518060 China; 3 Department of Physics and Materials Science, City University of Hong Kong, Tat Chee Avenue, Kowloon, Hong Kong, 999077 China

Correction to: *Nature Communications* 10.1038/ncomms12967, published online 30 September 2016.

This Article contains an error in Fig. 4c and Supplementary Figs. 5b and 6d.

In Fig. 4c, the panel representing the MCF7 cells at 20 ppm was inadvertently duplicated. The correct version of Fig. 4c is



In Supplementary Fig. 5b, the two SEM images for “After irradiation” and “1 week” were not measured on the same batch as the other two SEM images in the figure representing “4 weeks” and “8 weeks”. The correct version of Supplementary Fig. 5b is



In Supplementary Fig. 6d, the panel representing BPQDs/PLGA NSs at 20 ppm was inadvertently duplicated . The correct version of Supplementary Fig. 6d is



The errors hasve not been corrected in the PDF or HTML versions of the Article.

