# Supplementary material for: Biodegradable black phosphorus-based nanospheres for in vivo photothermal cancer therapy
Source: Nat Commun. 2016 Sep 30;7:12967. doi: 10.1038/ncomms12967 (PMC5056460; doi:10.1038/ncomms12967)
Supplement: Supplementary Information — Supplementary Figures 1-15, Supplementary Table 1 and Supplementary References. [file ncomms12967-s1.pdf]

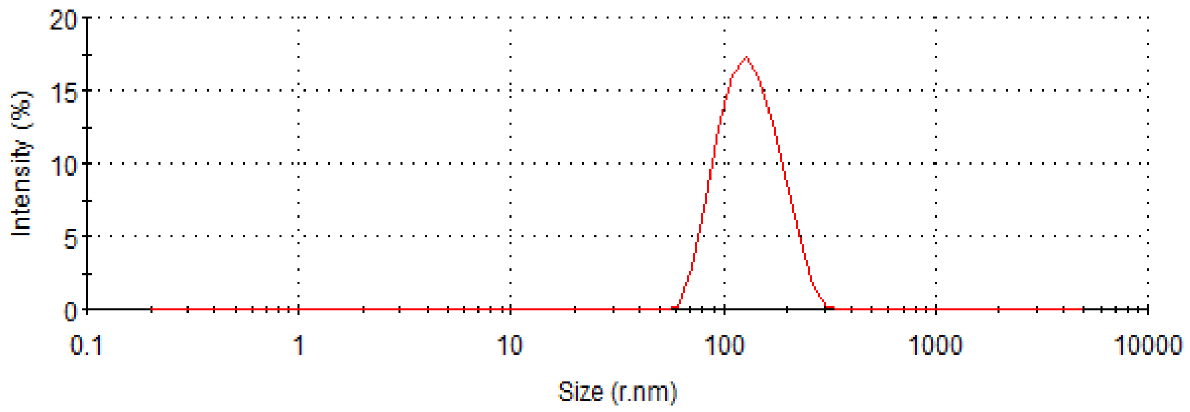

Supplementary Figure 1 | Measurement of the hydrodynamic size of BPQDs/PLGA NSs. Average hydrodynamic size of the BPQDs/PLGA NSs.

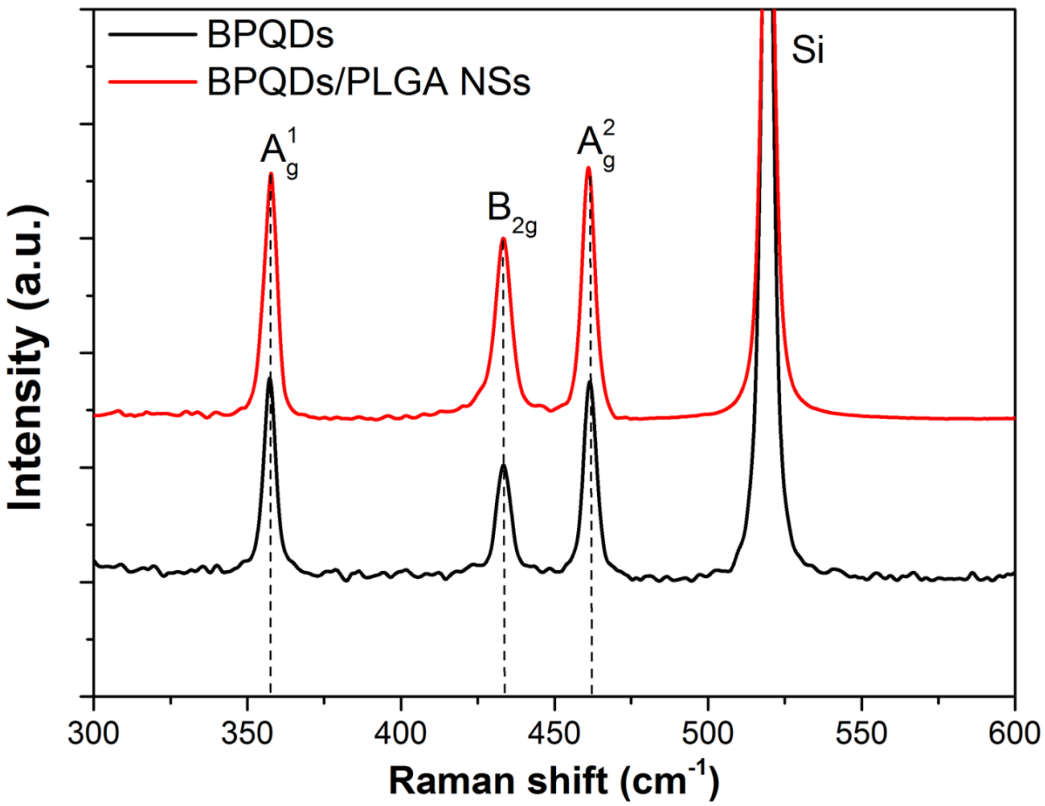

Supplementary Figure 2 | Raman scattering. Raman scattering spectra of the BPQDs and BPQDs/PLGA NSs.

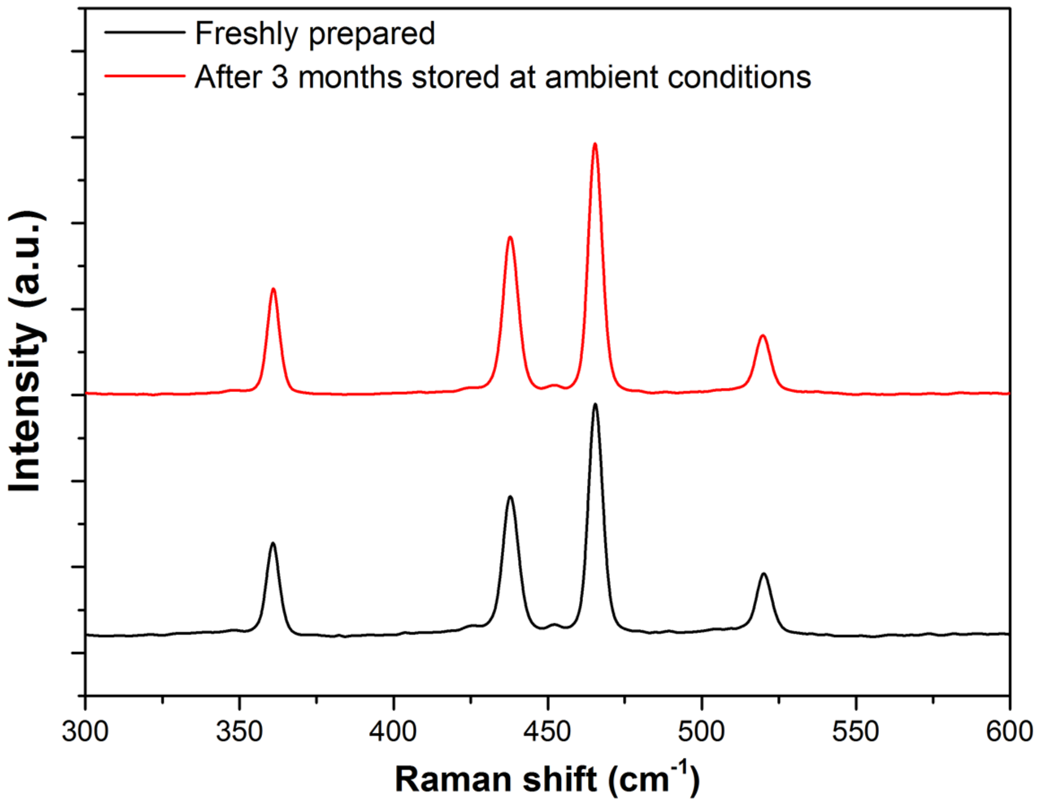

Supplementary Figure 3 | Raman scattering. Raman scattering spectra of the BPQDs/PLGA NSs prepared freshly and after storing under ambient conditions for 3 months.

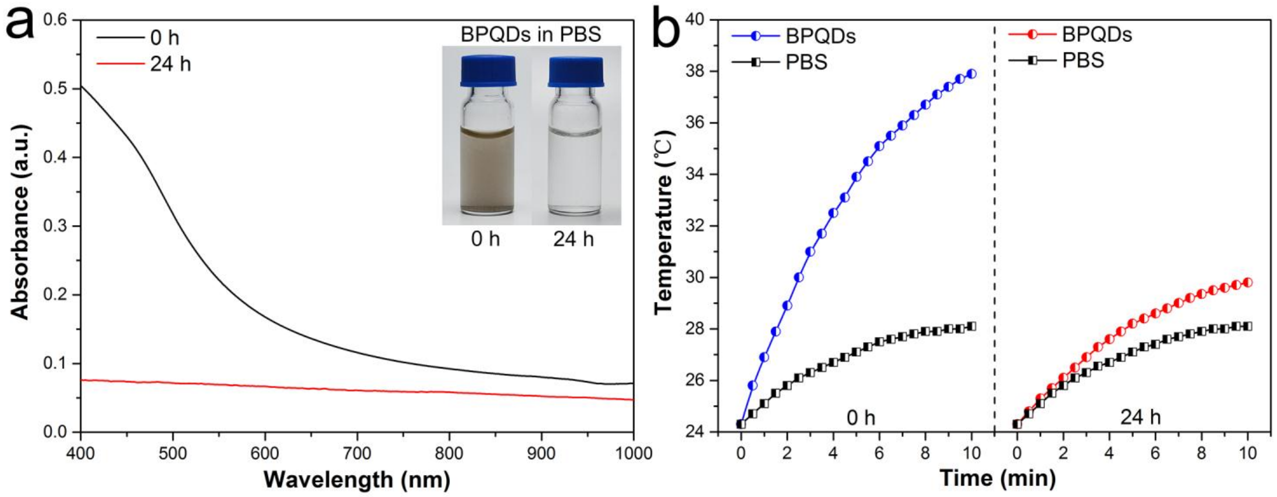

Supplementary Figure 4 | Biodegradation performance of BPQDs in PBS. (a) Absorption spectra of the BPQDs dispersed in PBS for 0 and 24 h with the corresponding photographs shown in the inset. (b) Photothermal heating curves of the BPQDs dispersed in PBS for 0 and 24 h.

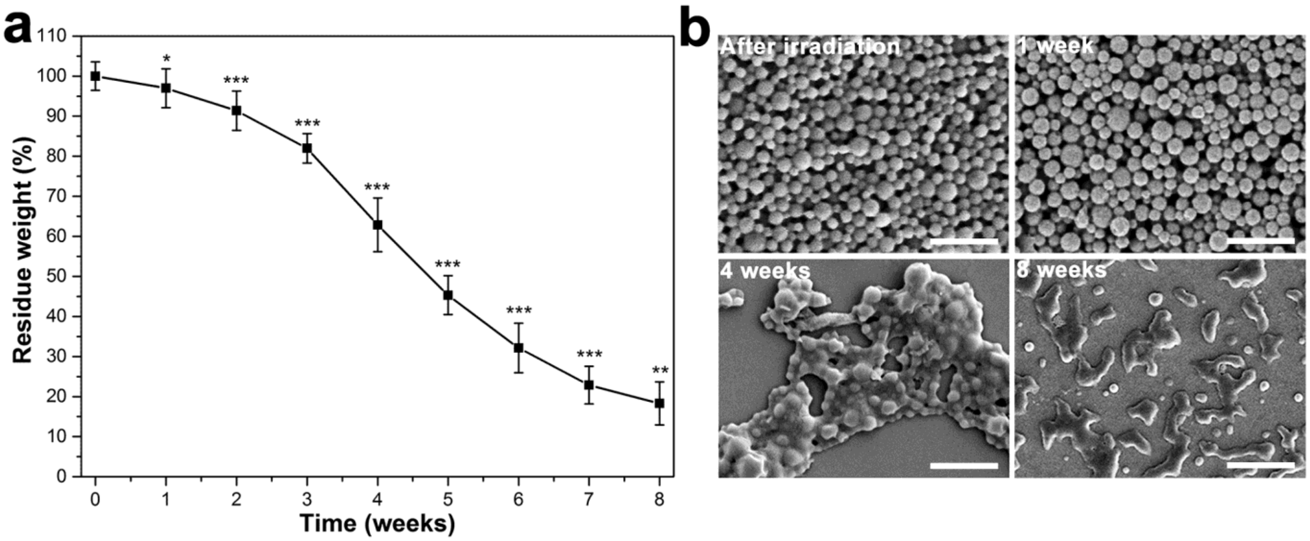

Supplementary Figure 5 | Biodegradation performance in PBS of BPQDs/PLGA NSs after irradiation by the 808 nm laser (1 W cm^{-2}) for 10 min. (a) Residual weight of the NSs irradiated and degraded in PBS as a function of time (n=5; $^{*}P < 0.05$, $^{**}P < 0.01$, $^{***}P < 0.001$; ANOVA). (b) SEM images of the NSs irradiated and then degraded in PBS for 0, 1, 4, and 8 weeks (scale bars, 500 nm).

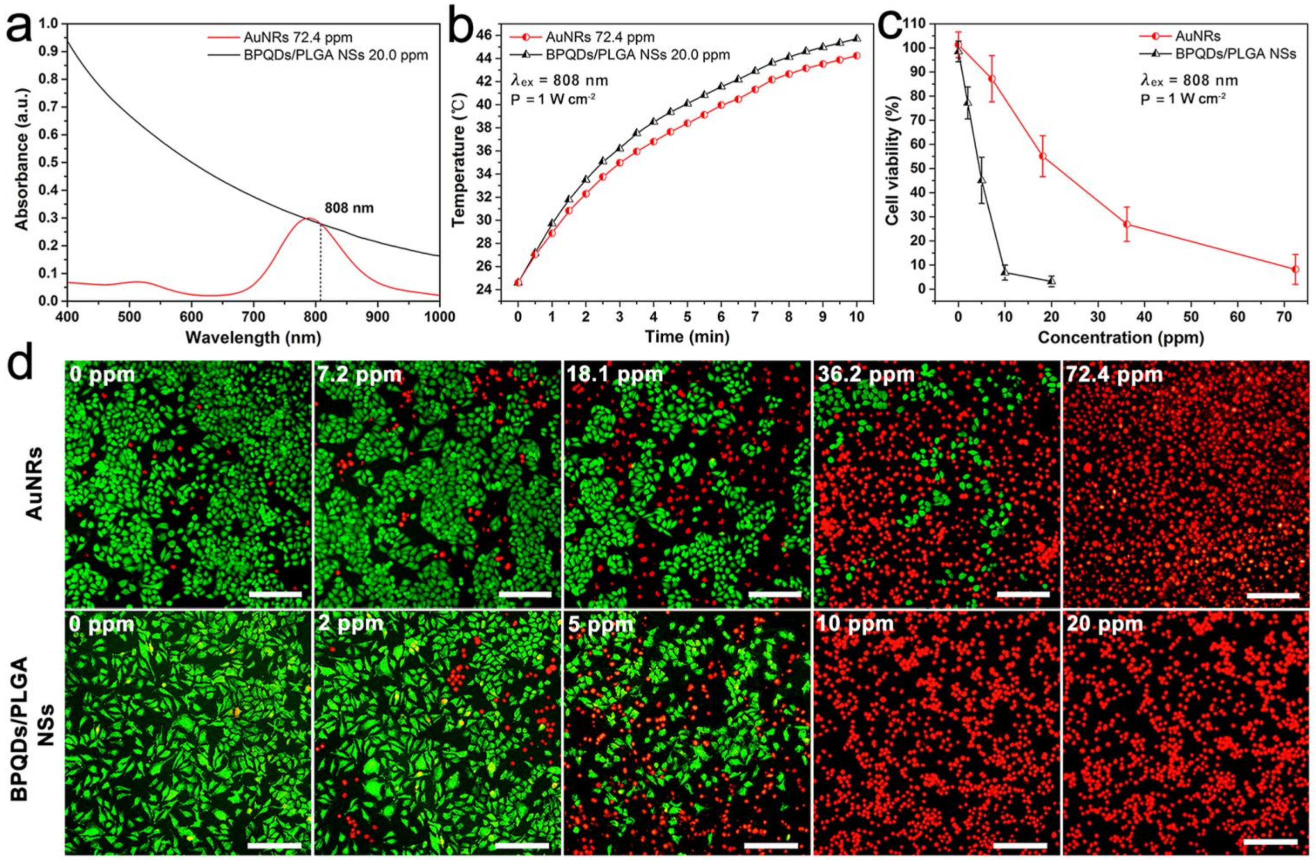

Supplementary Figure 6 | Comparison of photothermal performance between the BPQDs/PLGA NSs and AuNRs. (a) Absorption spectra and (b) Photothermal heating curves of the BPQDs/PLGA NSs (20.0 ppm BPQDs) and AuNRs (72.4 ppm) with the same absorption at 808 nm. The AuNRs as the positive control are synthesized in an aqueous solution by a common seed-mediated growth method reported previously[1,2] and BSA is used to conjugate with the AuNRs using a method described previously[2,3]. (c) Relative viability of the MCF7 cells after incubation with BPQDs/PLGA NSs and AuNRs with different concentrations (same absorption at 808 nm) for 4 h and irradiated with the 808 nm laser (1 W cm^{-2}) for 10 min. (d) Corresponding fluorescence images (scale bars, 100 μm for all panels) of the cells stained with calcein AM (live cells, green fluorescence) and PI (dead cells, red fluorescence).

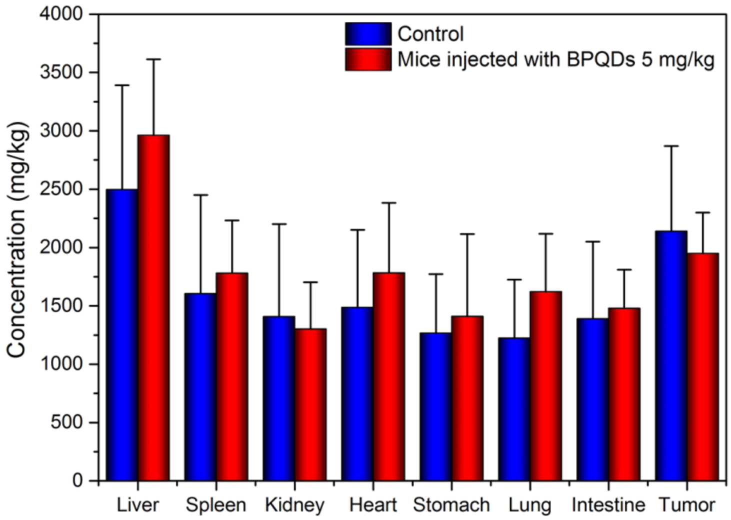

Supplementary Figure 7 | *In vivo* biodistribution analysis of BPQDs. The concentrations of P in tumor and major organs of MCF7 breast tumor-bearing mice without or with BPQDs injection (5 mg kg^{-1}) are determined by ICP-AES. Since the original P concentrations in the organs are much larger than the injection dose, no meaningful difference can be observed from the P concentrations in all the organs after injection of the BPQDs.

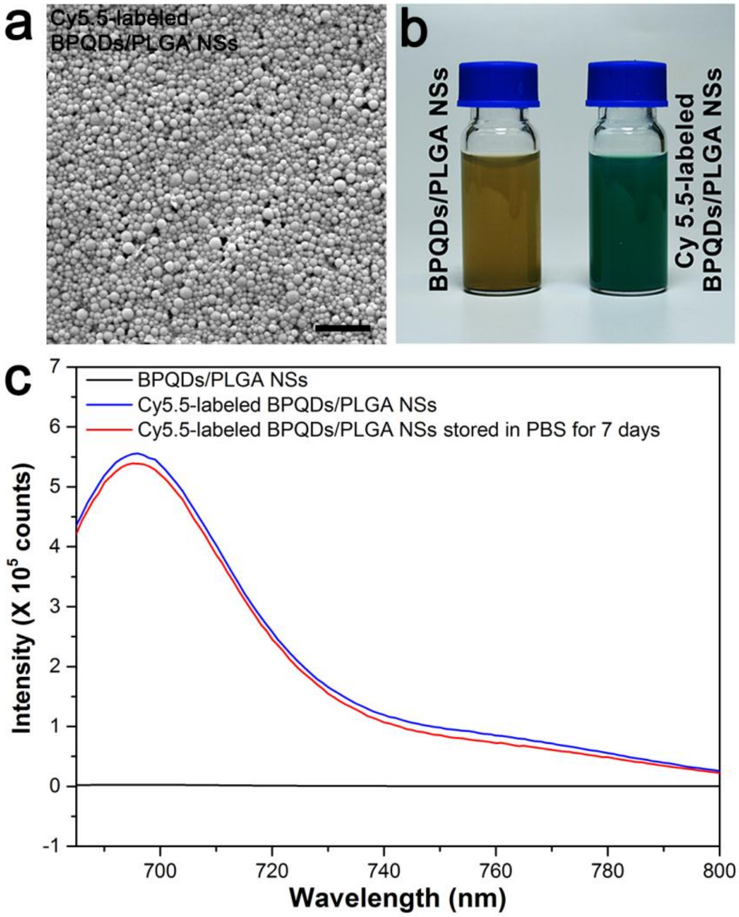

Supplementary Figure 8 | Characterization of the Cy5.5-labeled BPQDs/PLGA NSs. (a) SEM image of the Cy5.5-labeled BPQDs/PLGA NSs (scale bar, 1 μm). (b) Photographs of the BPQDs/PLGA NSs and Cy5.5-labeled BPQDs/PLGA NSs. (c) Emission spectra (λ_{ex} = 675 nm) of the Cy5.5-labeled BPQDs/PLGA NSs before and after storage in PBS for 7 days. No fluorescence can be observed from the BPQDs/PLGA NSs without Cy5.5 labeling.

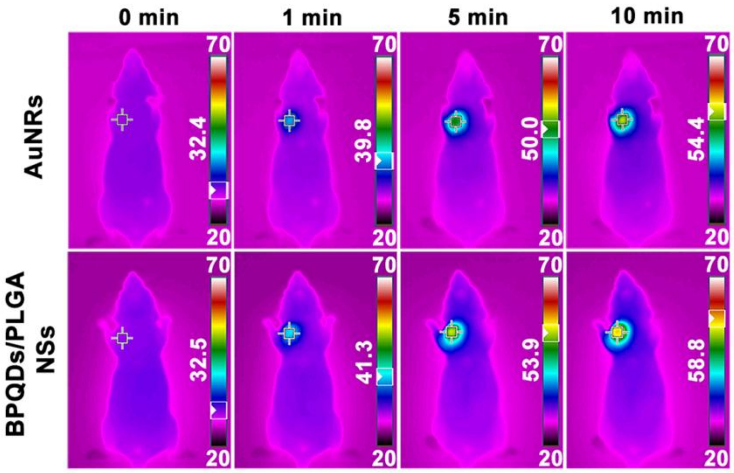

Supplementary Figure 9 | Photothermal performance comparison of the BPQDs/PLGA NSs and AuNRs for *in vivo* tumor ablation. Infrared thermographic maps of the tumor-bearing nude mice irradiated by the 808 nm laser (1 W cm^{-2}) at 24 h after separate intravenous injection with 100 μL of BPQDs/PLGA NSs (1 mg BP mL^{-1}) and AuNRs (3.62 mg mL^{-1}) with the color bar referring to the relative temperature.

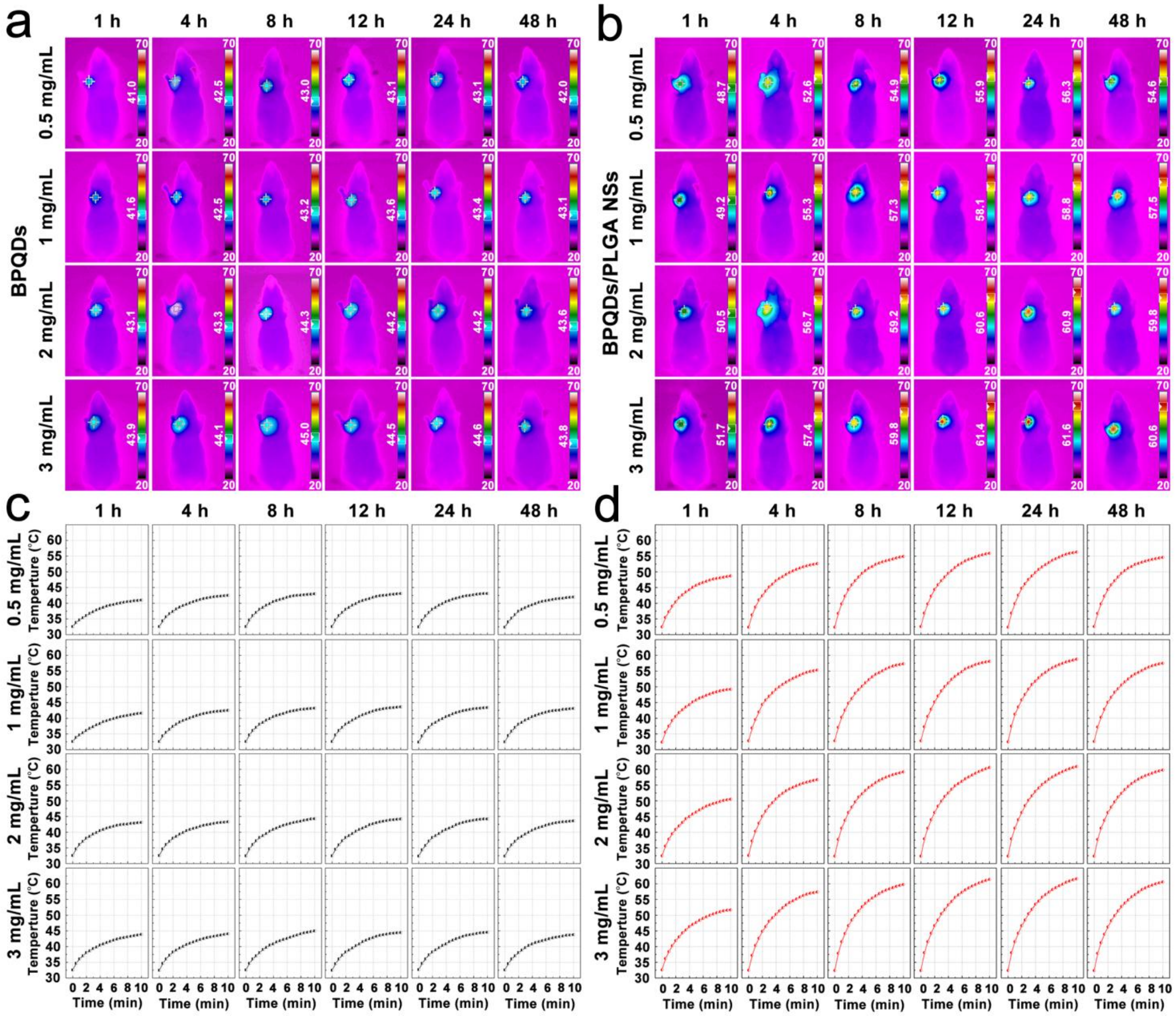

Supplementary Figure 10 | Photothermal performance comparison between the BPQDs/PLGA NSs and bare BPQDs for *in vivo* tumor ablation. (a, b) Infrared thermographic maps and (c, d) Time-dependent temperature increase in the tumor-bearing nude mice after separate intravenous injection of the BPQDs and BPQDs/PLGA NSs with different concentrations (0.5, 1, 2 and 3 mg BP mL^{-1}) and irradiation with the 808 nm laser (1 W cm^{-2}) at different time points (1, 4, 8, 12, 24 and 48 h) after injection. The color bars refer to the relative temperature.

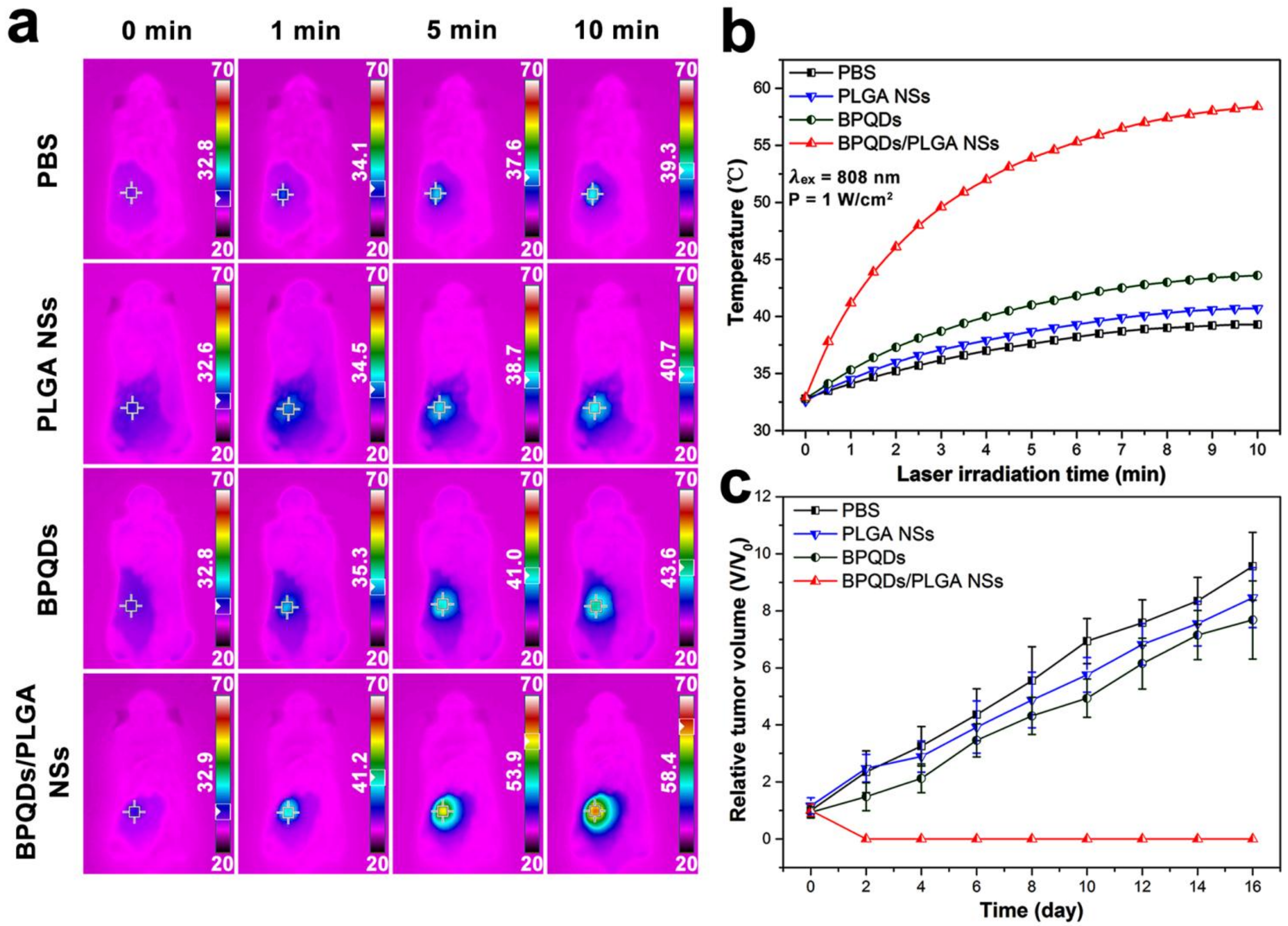

Supplementary Figure 11 | *In vivo* photothermal cancer therapy in the B16 tumor-bearing mice. (a) Infrared thermographic maps and (b) Time-dependent temperature increase in the B16 tumor-bearing nude mice irradiated by the 808 nm laser (1 W cm^{-2}) at 24 h after separate intravenous injection with 100 μL of PBS, PLGA NSs, BPQDs (1 mg mL^{-1}) and BPQDs/PLGA NSs (1 mg BP mL^{-1}) with the color bar referring to the relative temperature. (c) Growth curves of B16 tumor in different groups of mice treated with 100 μL of PBS, PLGA NSs, BPQDs (1 mg mL^{-1}), and BPQDs/PLGA NSs (1 mg BP mL^{-1}) with the laser irradiation.

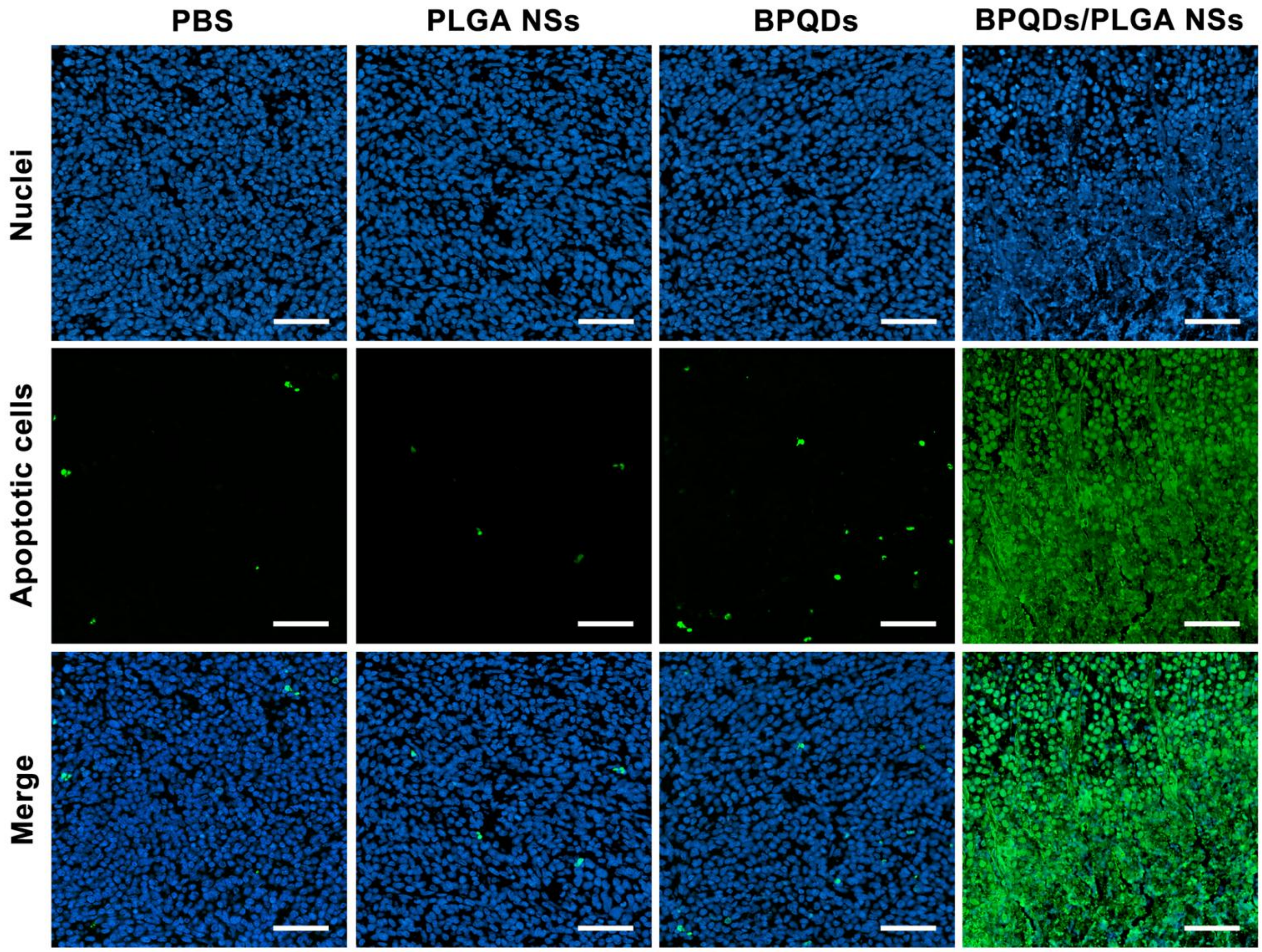

Supplementary Figure 12 | Apoptosis detection after *in vivo* photothermal cancer therapy. Fluorescence microscopy images (scale bars, 50 μm for all panels) of the apoptotic cells in the tumor tissues of the Balb/c nude mice irradiated by the 808 nm laser (1 W cm^{-2}) at 24 h after separate intravenous injection with 100 μL of PBS, PLGA NSs, BPQDs (1 mg mL^{-1}) and BPQDs/PLGA NSs (1 mg BP mL^{-1}). The apoptotic cells are labeled with FITC using the TUNEL assay (shown in green) and the nuclei are stained with DAPI (shown in blue).

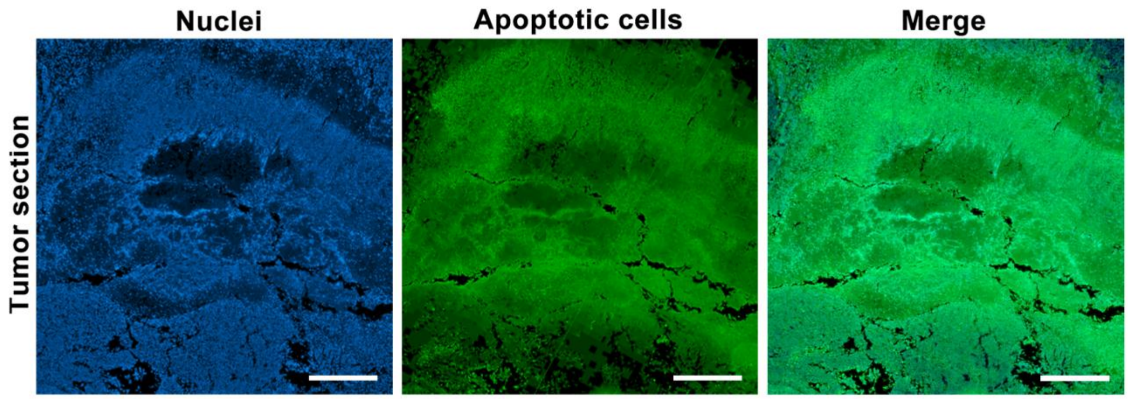

Supplementary Figure 13 | Apoptosis examination at the macro-organizational level. Fluorescence microscopy images (scale bars, 500 μm) of the apoptotic cells in the tumor sections at the macro-organizational level (about 10 mm^2) acquired from the Balb/c nude mice irradiated by the 808 nm laser (1 W cm^{-2}) at 24 h after intravenous injection with 100 μL of BPQDs/PLGA NSs (1 mg BP mL^{-1}). The apoptotic cells are labeled with FITC using the TUNEL assay (shown in green) and the nuclei are stained with DAPI (shown in blue).

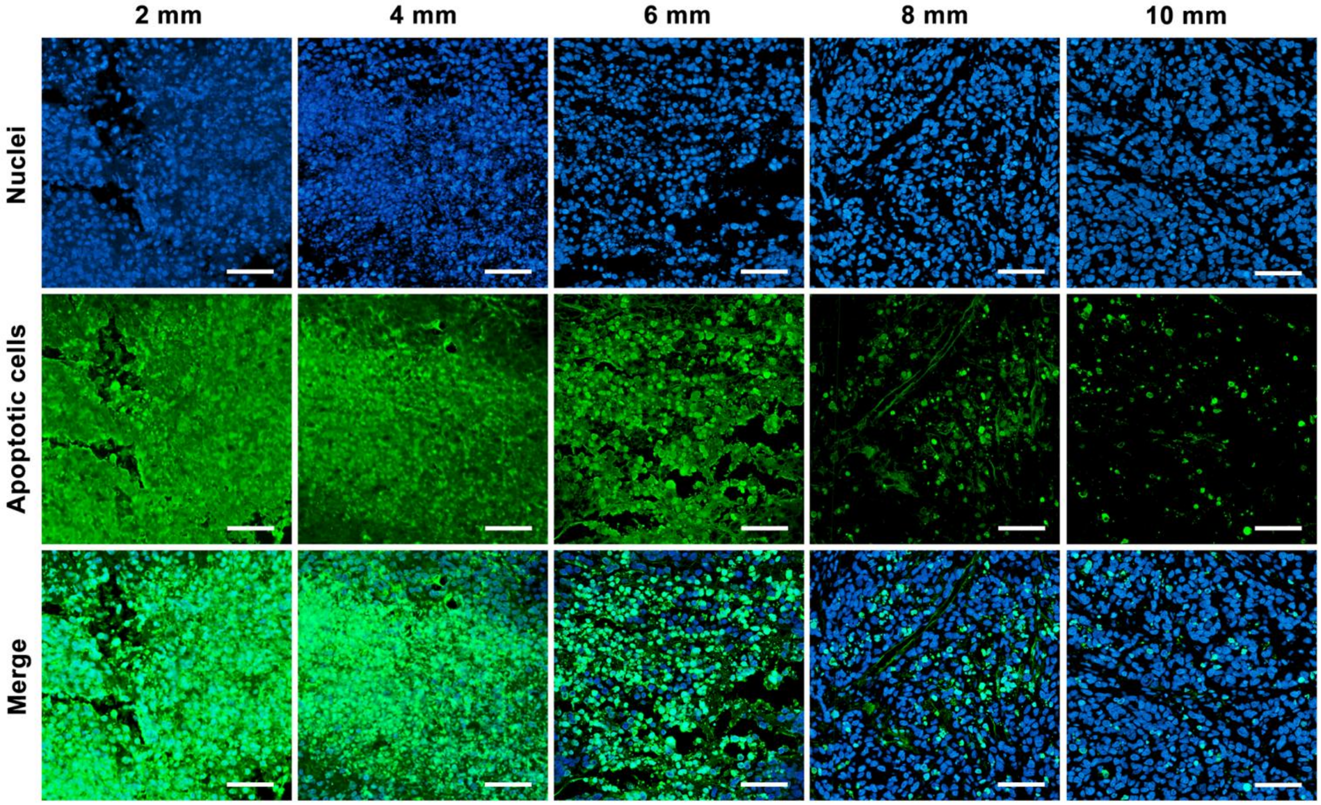

Supplementary Figure 14 | Apoptosis detection of tumor sections at different depths. Fluorescence microscopy images (scale bars, 50 μm for all panels) of the apoptotic cells in the tumor sections at different depths of the Balb/c nude mice irradiated by the 808 nm laser (1 W cm^{-2}) at 24 h after intravenous injection with 100 μL BPQDs/PLGA NSs (1 mg BP mL^{-2}). The apoptotic cells are labeled with FITC using the TUNEL assays (shown in green) and the nuclei are stained with DAPI (shown in blue).

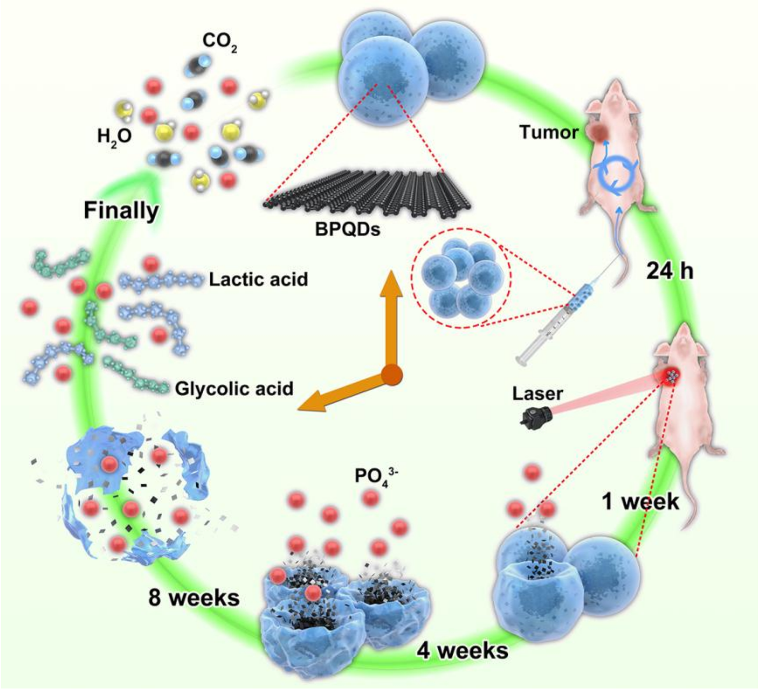

Supplementary Figure 15 | Schematic representation of the *in vivo* degradation process of the BPQDs/PLGA NSs inside the body.

		PBS					PLGA NSs				
Time	(mm)	No.1	No.2	No.3	No.4	No.5	No.1	No.2	No.3	No.4	No.5
0 d	Length	10.3	7.5	8.7	7.5	8.9	8.3	8.1	8.5	9.0	7.9
	Width	7.1	6.6	7.4	6.7	6.4	6.7	6.6	7.6	7.6	7.1
2 d	Length	12.8	9.1	10.5	9.3	12.4	9.1	9.4	9.6	10.5	9.5
	Width	9.4	8.8	9.7	9.0	8.7	7.7	7.4	8.7	8.6	8.0
4 d	Length	13.1	10.7	11.1	11.0	12.2	9.9	9.8	11.0	11.5	10.1
	Width	10.3	9.3	10.5	9.4	9.9	7.8	7.7	9.1	9.2	8.8
6 d	Length	14.7	12.0	12.2	12.9	14.0	10.8	10.7	12.3	12.8	11.1
	Width	11.1	10.5	11.7	10.5	11.0	8.3	8.1	10.1	10.2	9.6
8 d	Length	15.2	12.6	13.3	13.4	14.7	13.3	13.3	14.3	14.7	13.2
	Width	12.5	11.7	12.6	11.7	12.4	10.3	10.2	11.5	11.6	11.3
10 d	Length	15.7	15.2	14.6	15.2	15.6	15.5	15.4	16.6	16.3	15.7
	Width	14.0	12.8	13.8	13.1	13.7	12.3	12.1	12.5	12.7	12.6
12 d	Length	16.1	15.8	15.3	15.7	15.9	16.3	16.2	17.2	17.2	16.1
	Width	15.2	13.7	14.7	13.9	14.9	12.7	12.6	13.5	13.7	13.5
14 d	Length	16.6	16.5	15.9	16.5	16.4	16.9	16.7	17.6	17.8	16.8
	Width	15.5	14.2	15.2	14.4	15.4	13.2	13.1	14.0	14.2	13.9
16 d	Length	18.2	17.3	16.9	17.1	17.7	17.5	17.6	17.9	18.3	17.3
	Width	16.2	14.6	15.9	14.9	16.1	13.6	13.5	14.9	15.0	14.8
		BPQDs					BPQDs/PLGA NSs				
Time	(mm)	No.1	No.2	No.1	No.2	No.1	No.2	No.1	No.2	No.1	No.2
0 d	Length	7.9	8.4	7.9	9.3	8.6	8.6	8.9	8.0	7.6	8.0
	Width	6.8	6.2	6.5	7.4	7.2	7.2	7.1	6.8	6.9	7.0
2 d	Length	9.9	9.4	8.4	10.5	10.4	0.0	0.0	0.0	0.0	0.0
	Width	7.9	6.7	7.3	8.5	8.4	0.0	0.0	0.0	0.0	0.0
4 d	Length	10.5	9.7	9.2	11.7	11.6	0.0	0.0	0.0	0.0	0.0
	Width	8.1	6.9	7.5	8.8	8.7	0.0	0.0	0.0	0.0	0.0
6 d	Length	12.7	12.0	12.1	13.8	13.7	0.0	0.0	0.0	0.0	0.0
	Width	9.2	8.5	8.7	10.2	10.2	0.0	0.0	0.0	0.0	0.0
8 d	Length	13.7	13.3	13.6	14.4	14.5	0.0	0.0	0.0	0.0	0.0
	Width	10.4	9.7	9.8	11.2	11.1	0.0	0.0	0.0	0.0	0.0
10 d	Length	15.1	15.9	15.9	16.5	16.1	0.0	0.0	0.0	0.0	0.0
	Width	11.7	10.8	11.1	12.2	12.2	0.0	0.0	0.0	0.0	0.0
12 d	Length	17.1	17.6	16.7	17.7	17.0	0.0	0.0	0.0	0.0	0.0
	Width	12.4	11.5	12.0	13.3	13.3	0.0	0.0	0.0	0.0	0.0
14 d	Length	17.5	17.7	17.5	17.9	17.4	0.0	0.0	0.0	0.0	0.0
	Width	13.1	12.2	12.5	13.8	13.8	0.0	0.0	0.0	0.0	0.0
16 d	Length	17.6	18.0	17.7	18.2	17.5	0.0	0.0	0.0	0.0	0.0
	Width	13.4	12.5	12.7	14.8	14.8	0.0	0.0	0.0	0.0	0.0

Supplementary Table 1 | Raw measurements for the tumor volumes of MCF7 breast tumor in different groups of nude mice treated with PBS, PLGA NSs, BPQDs and BPQDs/PLGA NSs.

Supplementary References

1. Shao, J. *et al*. PLLA nanofibrous paper-based plasmonic substrate with tailored hydrophilicity for focusing SERS detection. *ACS Appl. Mater. Interfaces* **7**, 5391-5399 (2015).
2. Li, Z. *et al*. Small gold nanorods laden macrophages for enhanced tumor coverage in photothermal therapy. *Biomaterials* **74**, 144-154 (2016).
3. Ali, M. R. K., Panikkanvalappil, S. R., & El-Sayed, M. A. Enhancing the efficiency of gold nanoparticles treatment of cancer by increasing their rate of endocytosis and cell accumulation using rifampicin. *J. Am. Chem. Soc.* **136**, 4464-4467 (2014).
